# Differential regulation of C5a receptor 1 in innate immune cells during the allergic asthma effector phase

**DOI:** 10.1371/journal.pone.0172446

**Published:** 2017-02-23

**Authors:** Fanny Ender, Anna V. Wiese, Inken Schmudde, Jing Sun, Tillman Vollbrandt, Peter König, Yves Laumonnier, Jörg Köhl

**Affiliations:** 1 Institute for Systemic Inflammation Research, University of Lübeck, Lübeck, Germany; 2 Institute for Anatomy, University of Lübeck, Lübeck, Germany; 3 Cell Analysis Core, University of Lübeck, Lübeck, Germany; 4 Division of Immunobiology, Cincinnati Children’s Hospital Medical Center and University of Cincinnati College of Medicine, Cincinnati, Ohio, United States of America; Technische Universitat Dresden, GERMANY

## Abstract

C5a drives airway constriction and inflammation during the effector phase of allergic asthma, mainly through the activation of C5a receptor 1 (C5aR1). Yet, C5aR1 expression on myeloid and lymphoid cells during the allergic effector phase is ill-defined. Recently, we generated and characterized a floxed green fluorescent protein (GFP)-C5aR1 knock-in mouse. Here, we used this reporter strain to monitor C5aR1 expression in airway, pulmonary and lymph node cells during the effector phase of OVA-driven allergic asthma. C5aR1 reporter and wildtype mice developed a similar allergic phenotype with comparable airway resistance, mucus production, eosinophilic/neutrophilic airway inflammation and Th2/Th17 cytokine production. During the allergic effector phase, C5aR1 expression increased in lung tissue eosinophils but decreased in airway and pulmonary macrophages as well as in pulmonary CD11b^+^ conventional dendritic cells (cDCs) and monocyte-derived DCs (moDCs). Surprisingly, expression in neutrophils was not affected. Of note, moDCs but not CD11b^+^ cDCs from mediastinal lymph nodes (mLN) expressed less C5aR1 than DCs residing in the lung after OVA challenge. Finally, neither CD103^+^ cDCs nor cells of the lymphoid lineage such as Th2 or Th17-differentiated CD4^+^ T cells, B cells or type 2 innate lymphoid cells (ILC2) expressed C5aR1 under allergic conditions. Our findings demonstrate a complex regulation pattern of C5aR1 in the airways, lung tissue and mLN of mice, suggesting that the C5a/C5aR1 axis controls airway constriction and inflammation through activation of myeloid cells in all three compartments in an experimental model of allergic asthma.

## Introduction

Allergic asthma is one of the most prevalent diseases of the western world. It develops in genetically susceptible individuals as a chronic inflammatory disorder of the upper airways leading to recurrent episodes of wheezing, breathlessness, chest tightness, and coughing. Allergic asthma is characterized by airway hyperresponsiveness (AHR), inflammation, increased mucus and allergen-specific immunoglogulin (Ig) E production, which is mainly driven by maladapative T helper (Th) 2 and Th17 cytokines [1].

Air-born allergens can cleave C3 or C5 directly through their protease activity resulting in the generation of C3a and C5a *in vitro* [2] or *in vivo* during experimental and clinical allergic asthma [3, 4]. It is well appreciated that the complement cleavage product C5a regulates development of allergic asthma during allergen sensitization and the effector phase [5]. Genetic ablation or pharmacological targeting of C5 [6, 7] or C5aR1 [3, 8, 9] during allergen sensitization resulted in aggravation of the allergic asthma phenotype, suggesting that C5aR1 protects from the development of allergic asthma. In contrast, blockade of the C5aR1 signaling during the effector phase decreased the asthmatic phenotype [10–12], demonstrating that C5a is pro-allergic in established asthma.

Several pulmonary immune and stromal cells express C5aR1 at steady state [5]. More specifically, C5aR1 expression has been described in myeloid and plasmacytoid DCs (pDCs) [3]. Recently, more sophisticated gating strategies were used to phenotypically characterize pulmonary immune cell subsets [13, 14] allowing a better mapping of C5aR1 expression in lung DC populations [15]. Among the four DC subsets present in the lung, only the CD11b^+^ conventional (c)DCs and the monocyte-derived (mo)DCs express C5aR1 [15, 16]. In moDCs, C5aR1 expression has been described as a specific marker, at least in C57BL/6 mice [16].

C5aR1 expression has been also observed in neutrophils [17], eosinophils [18], and alveolar macrophages [19]. GFP-C5aR1 reporter mice confirmed the C5aR1 expression in macrophages, neutrophils [15, 20], eosinophils and DC subsets [15]. In contrast, the expression of C5aR1 by CD4^+^ T cells is still controversial [15, 20, 21].

While the expression pattern of C5aR1 in pulmonary cells at steady state is relatively clear, the regulation of C5aR1 expression under allergic asthma conditions during the effector phase remains elusive. In an OVA-driven allergic asthma model in the rat, the mRNA encoding for C5aR1 was reported to increase in the whole lung upon OVA challenges [10]. Antibody-targeting approaches revealed that this increase was not due to upregulation of C5aR1 in the parenchymal cell compartment but in infiltrating leukocytes [22].

Here, we performed a precise expression profiling of C5aR1 during the effector phase of experimental allergic asthma. We used WT and floxed GFP-C5aR1 reporter mice (GFP-C5aR1^flox/flox^) [15] in a model of OVA-driven allergic asthma and assessed C5aR1 expression in myeloid and lymphoid cells isolated from the airways, lung tissue and mLN. Our data demonstrate that C5aR1 is expressed and differentially regulated in the myeloid but not in the lymphoid compartment.

## Materials and methods

### Mice

GFP-C5aR1^flox/flox^ mice were described previously [15]. WT control mice were obtained from Janvier. All mice were bred and maintained at the University of Lübeck specific pathogen-free facility and used for experiments at 8–12 weeks of age. Animal care was provided in accordance with German rights. This study was reviewed and approved by the Schleswig-Holstein state authorities (Nr. V242-30397/2016 (56-5/16)).

### Experimental ovalbumin (OVA)-driven allergic asthma model

The OVA-induced asthma model was performed as described previously [23] with minor modifications (S1 Fig). Briefly, mice were immunized by intra-peritoneal (i.p.) injection with OVA-Alum (200μg/2mg) on days 0 and 7 and challenged intratracheally (i.t.) with 1.5% OVA (in 50 μl PBS) on days 14, 16, 18, and 20. On day 21 (24 h after the last i.t. administration), AHR was determined and tissue samples were harvested for further analysis.

### Determination of airway hyperresponsiveness

Mice were anaesthetized by i.p. injection of 50 μl Ketavet/Rompun (76 mg/ml and 4.8 mg/ml respectively, Pfizer/Bayer). Muscle relaxation was induced by administration of 50 μl of Esmeron (10 mg/ml, Organon). Airway hyperresponsiveness (AHR) was measured in anesthetized mice that were mechanically ventilated using a FlexiVent (SciReq) system as described [24]. Aerosolized Acetyl-β-Methyl-Choline (methacholine) (0, 2.5, 5, 10, 25, and 50 mg/ml; Sigma-Aldrich) was generated by an ultrasonic nebulizer and delivered in-line through the inhalation port for 10 seconds. Airway resistance was measured two minutes later.

### Collection of Bronchoalveolar Lavage (BAL) fluid and differential cell counting

BAL fluid samples were obtained by cannulating the trachea, injecting 1 ml of ice-cold PBS, and by subsequently aspirating the BAL fluid. After red blood cell lysis, BAL fluid cells were washed once in PBS and counted using a Neubauer chamber (Assistant, Germany). Frequencies of BAL fluid cells were determined by flow cytometry. Cell numbers were calculated using cell specific frequency of total and total cell counts/ml.

### Antibodies

Monoclonal phycoerythrin (PE)-labeled Ab against C5aR1/CD88 (20/70) was obtained from AbD Serotec, Brilliant Violet (BV) 421- or PE-labeled Abs against SiglecF (E50-2440), V450- or allophycocyanin (APC)-labeled Abs against Ly6G (1A8), were purchased from BD Biosciences. EFluor (eF)450-labeled Abs against CD19 (1D3), CD3e (145-2C11) or CD49b (DX5); APC-labeled Ab against CD11c (N418) and PE-cyanine (Cy)7-labeled Ab against CD4 (RM4-5) were obtained from eBioscience (Affimetrix). BV510-labeled Ab against CD11b (M1/70), PE-labeled Ab against CD64 (X54-5/7.1), APC-eF780–labeled Ab against MHC class II (M5/144.15.2), APC-labeled Ab against CD62L (MEL-14), BV421-labeled Ab against CD44 (IM7), and Peridinin chlorophyll protein—cyanine 5.5 (Per-CP–Cy5.5)–labeled Ab against CD103 (2E7) were all purchased from Biolegend. Per-CP–Cy5.5–labeled Ab against CD3 (17A2), CD5 (53–73), CD27 (LG.7F9), NK1.1 (PK136), TCRβ (H57-597), CD11b (MI/70); eF780–labeled Ab against CD11c (N418), B220 (RA3-6B2), CD49b (DX5); eF450–labeled Ab against CD25 (PC61.5); PE-labeled Ab against CD90.2 (30-H12); PE-cy5-labeled Ab against CD127 (A7R34) were purchased from eBioscience (Affimetrix) except anti-CD90.2 (BD Pharmingen)

### Lung cell isolation and flow cytometric analysis

Liberase TL (Roche) 0.25 mg/ml and DNaseI 0.5 mg/ml (Sigma-Aldrich) digests of the lungs were prepared to obtain single lung cell suspensions. Phenotypic characterization of cells was performed on a BD LSRII or an ARIA III flow cytometer using recently published gating strategies [13, 15]. ILC2 were identified as Lineage− cells lacking the expression of lineage markers associated with T cells (CD3, CD5, TCR and CD27), B cells (B220), macrophages (CD11b), DCs (CD11c) and natural killer (NK) cells (NK1.1, CD49b). These Lineage− cells expressed CD90.2 (alloantigen Thy-1), CD25 (α-chain of the receptor for IL-2) and CD127 (α-chain of the receptor for IL-7) [25].

### Lung histology

Lung histological staining, detection and quantification of mucus cell content were done as described previously [24]. Slides were stained either with hematoxylin and eosin (H/E), or periodic acid-Schiff (PAS). PAS positive and PAS negative airways were counted by light microscopy and the percentages of PAS positive airways were calculated to quantify mucus production.

### RNA isolation from CD4^+^CD44^+^CD62L^-^ T effector cells and real time PCR

CD4^+^CD44^+^CD62L^-^T effector cells were sorted using a BD FACS ARIA III. CD4^+^CD44^+^CD62L^-^ effector T cells were sorted using a BD FACS ARIA III. RNA were isolated using Trizol reagent according to the manufacturer’s instructions (Invitrogen). Reverse transcription reaction of total RNA was performed after degradation of contaminating genomic DNA using DNase I (Fermentas), using a first strand cDNA synthesis kit (Revertaid Premium, Fermentas). Quantitative PCR was done using iQ Syber green (Biorad) on a CFX96 real-time PCR system (Biorad) using the specific primers (Eurofin). Differentiation of T helper (Th) cells was evaluated by amplifying Th-specific transcription factors: TBX21 (Th1) 5’-GGTGTCTGGGAAGCTGAGAG-3’ (sense) and 5’-ATCCTGTAATGGCTTGTGGG-3’ (antisense), GATA3 (Th2) 5’-GCCTGCGGACTCTACCATAA-3’ (sense) and 5’-AGGA TGTCCCTGCTCTCCTT-3’ (antisense), RORγT (Th17) 5’-CCGCTGAGAGGGCTTCAC-3’ (sense) and 5’-TGCAGGA GTAGGCCACATTACA-3’ (antisense) and FoxP3 (Treg) 5’-CCCATCCC CAGGAGTCTTG-3’ (sense) and 5’-ACCATGACTAGGGGCACTGTA-3’ (antisense). To determine the cytokine expression, we amplified IL-13 using 5’-CCTGGCTCTTGCTTGCCTT-3’ (sense) and 5’- GGTCTTGTGTGATGTTGCTCA-3’ (antisense) primers, and IL-17A using 5’- CTCCAGAAGGCCCTCAGACTAC-3’ (sense) and 5’- AGCTTTCCCTCCGCATTGACACAG-3’ (antisense) primers. In all samples, actin 5’-GCACCACACCTTCTACAATGAG-3’ (sense) and 5’-AAATAGCACAGCCTGGATAGCAAC-3’ (antisense) was used as an internal control. Real-time RT-PCR data were analyzed using CFX Manager Software 3.1 (Bio-Rad).

### Statistical analysis

Statistical analysis was performed using the GraphPad Prism version 5 (GraphPad Software, Inc.). Normal distribution of data was tested using the Kolmogorov-Smirnov and D'Agostino-Pearson tests, some after log transformation. When groups were normally distributed, statistical differences between two groups were analyzed by unpaired t test. Comparisons involving multiple groups were first analyzed by ANOVA followed by Tukey's test. When groups were not normally distributed, they were analyzed using a Mann-Whitney U (two groups), or an ANOVA on ranks (mutiple groups) followed by a Dunn's multiple comparison test. A p value < 0.05 was considered as * or § significant, p <0.01 as ** or §§ significant and p <0.001 as *** significant.

## Results

### Characterization of the allergic phenotype and C5aR1 expression in WT and GFP-C5aR1^flox/flox^ mice

**Fig 1:**
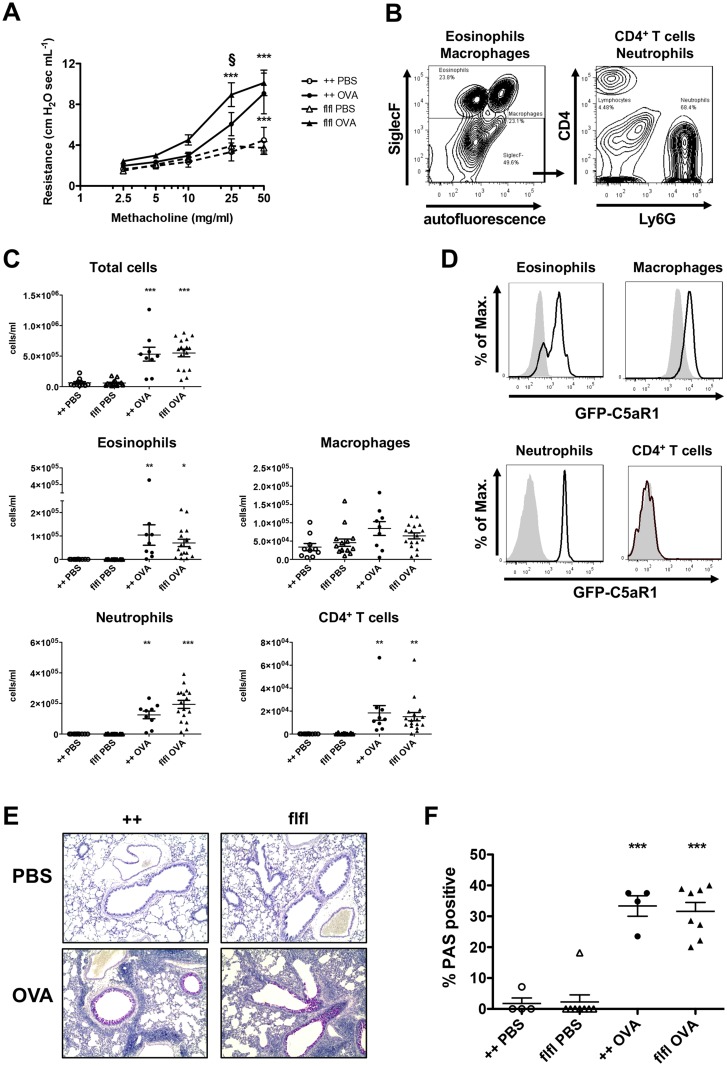
WT and GFP-C5aR1^flox/flox^ mice develop a similar allergic asthma phenotype. **(A)** AHR in response to i.t. administration of methacholine measured as airway resistance. Shown are dose response curves in PBS-treated controls or OVA-immunized mice from WT (++) or GFP-C5aR1^flox/flox^ (flfl) strains. Values shown are the mean ± SEM; n = 9–16 per group. **(B)** Gating strategy for the BAL fluid cell analysis. Cells were identified by flow cytometry using different markers to identify macrophages (SiglecF^+^autofluorescence^+^), eosinophils (SiglecF^+^autofluorescence^-^) neutrophils (SiglecF^-^Ly6G^+^CD4^-^), and T cells (SiglecF^-^Ly6G^-^CD4^+^). **(C)** Total and differential cell counts in BAL fluid of PBS-treated or OVA-immunized WT or GFP-C5aR1^flox/flox^ animals. Values shown are the mean ± SEM; n = 9–17 per group. **(D)** GFP/C5aR1 expression in eosinophils, macrophages, neutrophils and CD4^+^ T cells from BAL fluid of GFP-C5aR1 reporter mice in response to OVA; grey histogram: WT controls. **(E)** Histological examination of mucus production in the airways of PBS-treated or OVA-immunized WT or GFP-C5aR1^flox/flox^ animals. Sections were stained with PAS for mucus production (original magnification x 200). (**F)** Frequency of PAS-positive bronchi in PBS-treated or OVA-immunized WT or GFP-C5aR1^flox/flox^ animals. Mucus producing airways are plotted relative to all analysed airways. Values shown are the mean ± SEM; n = 4–8 per group. * indicates significant differences between the PBS and OVA treatment groups; § indicates significant differences between OVA-treated WT and GFP-C5aR1^flox/flox^ mice. * or § p < 0.05, ** p < 0.01, *** p <0.001.

C5aR1 expression in pulmonary immune cells under asthmatic conditions is ill-defined. Using floxed GFP-C5aR1 reporter mice, we recently described C5aR1 expression in lung neutrophils, eosinophils, macrophages, CD11b^+^ cDCs and moDCs but not in CD103^+^ cDCs under steady state conditions. Further, we observed C5aR1 expression in BAL macrophages [15]. Here, we monitored C5aR1-expression in myeloid and lymphoid cells in a model of OVA-driven allergic asthma (S1 Fig). Twenty-four hours after the final challenge, we determined airway resistance and airway inflammation. OVA-immunized WT and GFP-C5aR1^flox/flox^ mice suffered from a significant increase in AHR in comparison to PBS-treated controls (Fig 1A). GFP-C5aR1^flox/flox^ mice showed a higher airway resistance than WT animals at low methacholine concentrations. In contrast, both strains reached a similar maximum at 50 mg/ml methacholine. Upon PBS treatment, macrophage numbers were high in the BAL of WT and GFP-C5aR1^flox/flox^ mice (Fig 1C). Upon OVA stimulation, we observed a strong recruitment of neutrophils, eosinophils and lymphocytes into the bronchoalveolar compartment in both, WT and GFP-C5aR1^flox/flox^ mice. The most prominent cell populations were neutrophils and eosinophils. T cell numbers were much lower (Fig 1C).

Then, by measuring the GFP signal as a surrogate marker for C5aR1 expression, we determined C5aR1 expression in immune cells present in the airways after OVA-treatment. We found a strong C5aR1 expression in eosinophils, macrophages and neutrophils, but no C5aR1 expression in CD4^+^ T cells (Fig 1D). The increased production of mucus by goblet cells is another feature of allergic asthma (Fig 1E and 1F). Compared to PBS treated control mice, WT and GFP-C5aR1^flox/flox^ mice showed a significant increase in the frequency of mucus-positive airways in response to OVA challenge (Fig 1E). We found no differences between WT and GFP-C5aR1^flox/flox^ allergic asthma groups.

Collectively, our data show that WT and GFP-C5aR1^flox/flox^ mice develop a similar asthmatic phenotype with comparable airway inflammation, mucus production but somewhat higher airway sensitivity of the reporter mice towards low methacholine concentrations. Further, myeloid but not lymphoid-derived cells express C5aR1 in the airways.

### Similar accumulation of myeloid cells in lung tissue of WT and GFP-C5aR1^flox/flox^ mice

**Fig 2:**
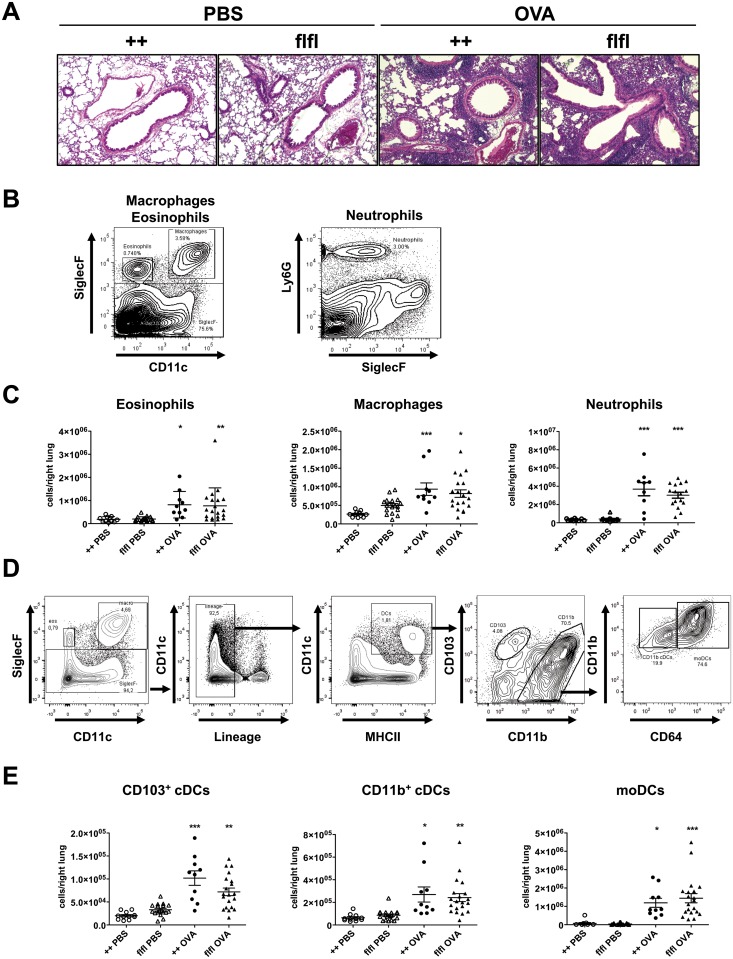
WT and GFP-C5aR1^flox/flox^ mice show a strong and similar pulmonary recruitment of inflammatory cells during the allergic effector phase. (**A)** Histological examination of airway inflammation. Sections were stained with H&E (original magnification x 200). The pictures are representative of 5 histological sections per treatment group. (**B**) Gating strategies used to identify eosinophils (SiglecF^+^CD11c^-^), macrophages (SiglecF^+^CD11c^+^) or neutrophils (Ly6G^+^SiglecF^-^) in lung tissue. **(C)** Differential cell counts of PBS-treated or OVA-immunized WT or GFP-C5aR1^flox/flox^ animals. Values shown are the mean ± SEM; n = 9–17 per group. (**D**) Gating strategy to identify DC subsets in lung tissue. Data shown represent the pulmonary cell composition of OVA-treated mice. Cells were first gated on SiglecF^-^ cells. Then lineage negative cells were excluded. Subsequently DCs were identified as CD11c^+^MHCII^+^ cells. These cells were further subdivided into CD103^+^CD11b^−^ or CD103^−^CD11b^+^cDCs. Within the latter population, we identified CD11b^+^CD64^-^ cDCs and CD11b^+^CD64^+^moDCs. (**E**) DC counts in lung cell suspensions of PBS-treated or OVA-immunized WT or GFP-C5aR1^flox/flox^ animals. Values shown are the mean ± SEM; n = 7–18 per group. * indicates significant differences between PBS or OVA-treated groups. * p < 0.05, ** p < 0.01, *** p <0.001.

Next, we performed histological examination of lungs from WT and GFP-C5aR1^flox/flox^ mice. We observed a strong recruitment of immune cells around blood vessels and airways (Fig 2A). To further characterize the composition of the infiltrate, lung cells were analyzed by flow cytometry (Fig 2B and 2D). Among the inflammatory cells, neutrophils were dominant in OVA-treated mice (Fig 2C). Also, the number of eosinophils and macrophages increased significantly in WT and GFP-C5aR1^flox/flox^ mice. We identified three pulmonary DC subsets in the lung (Fig 2D) [13]. At steady state, CD11b^+^ cDCs, CD103^+^ cDCs and a minor population of moDCs were present (Fig 2E). In response to OVA, the numbers of CD103^+^, CD11b^+^ cDCs and moDCs markedly increased (Fig 2E). Within the CD11b^+^ DC population about 20–30% were CD11b^+^ cDCs, 70–80% were moDCs in WT and GFP-C5aR1^flox/flox^ mice after OVA challenge. Overall, the accumulation of cDCs and moDCs was similar in both strains.

### Accumulation and differentiation of lymphoid cells in lung tissue of WT and GFP-C5aR1^flox/flox^ mice

**Fig 3:**
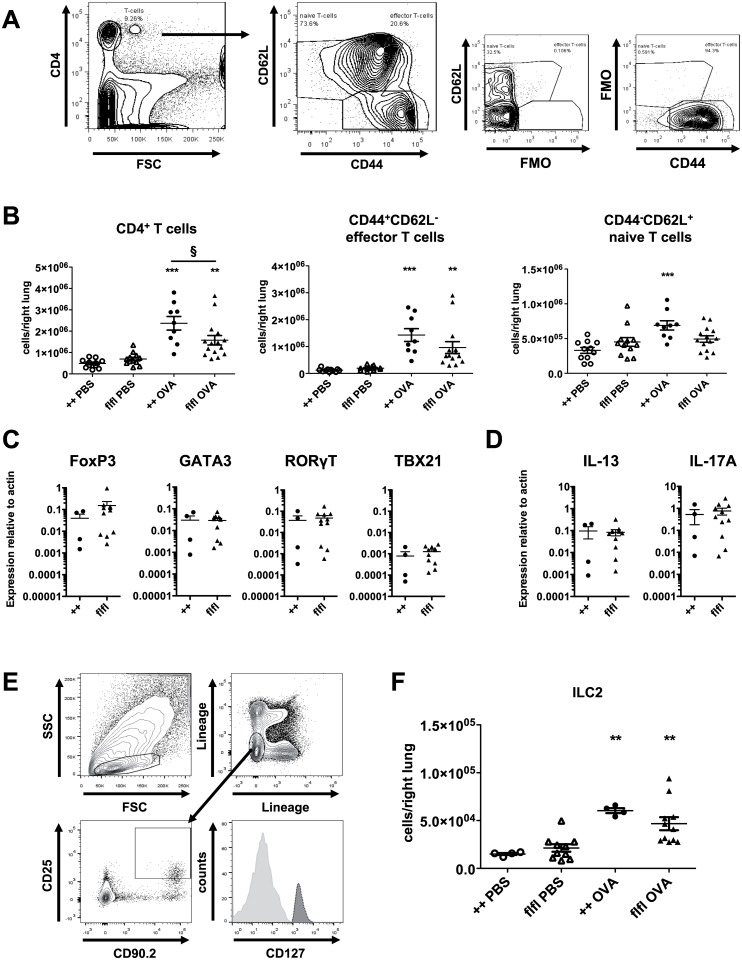
CD4^+^ T cell and ILC2 accumulation as well as CD4^+^ T cell differentiation in WT and GFP-C5aR1^flox/flox^ mice in the allergic effector phase. **(A)** Gating strategy to identify CD4^+^ T cells. T cells were subdivided into naive (CD44^-^CD62L^+^) and effector (CD44^-^CD62L^+^) T cells. **(B)** Recruitment of different T cell populations into the lungs of PBS-treated or OVA-immunized WT or GFP-C5aR1^flox/flox^ animals. Values shown are the mean ± SEM; n = 9–15 per group. **(C)** Expression of FoxP3 (Tregs), GATA3 (Th2), RORγT (Th17), and TBX21 (Th1) transcripts in sorted CD44^+^CD62L^-^ T cells. The abundance of transcripts was evaluated after reverse transcription by real-time PCR. Values shown are the mean abundance of target mRNA as compared to actin analyzed by Mann-Whitney test (n = 4–11). **(D)** Comparison of IL-13 and IL-17A mRNA expression levels in WT and GFP-C5aR1^flox/flox^ mice in sorted CD44^+^CD62L^-^ T cells as determined by real-time PCR. Values shown are the mean abundance of target mRNA as compared to actin analyzed by Mann-Whitney test (n = 4–11). **(E)** Gating strategy used to identify ILC2 in lung tissue (Lin^-^CD25^+^CD90.2^+^CD127^+^). **(F)** ILC2 cell numbers in lung tissue of PBS-treated or OVA-immunized WT or GFP-C5aR1^flox/flox^ animals. Values shown are the mean ± SEM; n = 4–11 per group. * indicates significant differences between PBS or OVA-treated groups; § indicates significant differences between WT and GFP-C5aR1^flox/flox^ OVA-treated groups. § p < 0.05, ** p < 0.01, *** p <0.001.

The number of CD4^+^ T cells increased significantly in WT and GFP-C5aR1^flox/flox^ mice under asthmatic conditions (Fig 3A and 3B). However, total CD4^+^ T cell numbers in GFP-C5aR1^flox/flox^ mice were lower than in WT mice. CD44^+^CD62^+^ effector T cells markedly increased in WT and C5aR1 reporter mice. Of note, we found that naïve CD44^-^CD62L^+^ T cells increased significantly in WT but remained at the same level in C5aR1^flox/flox^ mice in response to OVA-treatment.

Next, we determined the differentiation pattern of pulmonary effector T cells into Th1, Th2, Th17 and Treg subtypes in sorted CD44^+^CD62^-^ effector T-cells. We found 100-times higher levels of mRNA encoding for FoxP3, GATA3, RORγT than those encoding for *TBX21* (TBet) with no differences between cells from WT or GFP-C5aR1^flox/flox^ mice (Fig 3C). These findings demonstrate a clear Th2/Th17 commitment of most effector T cells. In addition, a strong population of Treg cells was present (Fig 3C). In line with the dominance of Th2/Th17 cells, we observed a high and similar abundance of IL-13 and IL-17A transcripts in CD44^+^C62L^-^ effector T cells from WT and GFP-C5aR1^flox/flox^ mice (Fig 3D).

In addition to Th2 effector cells, ILC2 drive the development of the allergic phenotype, in particular through production of IL-13 and IL-5 [26]. ILC2 were defined as lineage^-^CD25^+^CD90.2^+^CD127^+^ cells (Fig 3E). Lung isolates from WT and GFP-C5aR1^flox/flox^ mice showed a significant increase in ILC2 numbers in the OVA-treatment groups (Fig 3F) in WT and GFP-C5aR1^flox/flox^ animals.

Taken together, our data demonstrate that the accumulation but not the differentiation of total CD4^+^ T cells is impaired in GFP-C5aR1^flox/flox^ mice as compared with WT mice. In contrast to CD4^+^ T cells, the accumulation of ILC2 is not affected in GFP-C5aR1^flox/flox^ mice.

### Comparison of C5aR1 expression in myeloid and lymphoid cells from the alveolar, pulmonary and mLN compartments

**Fig 4:**
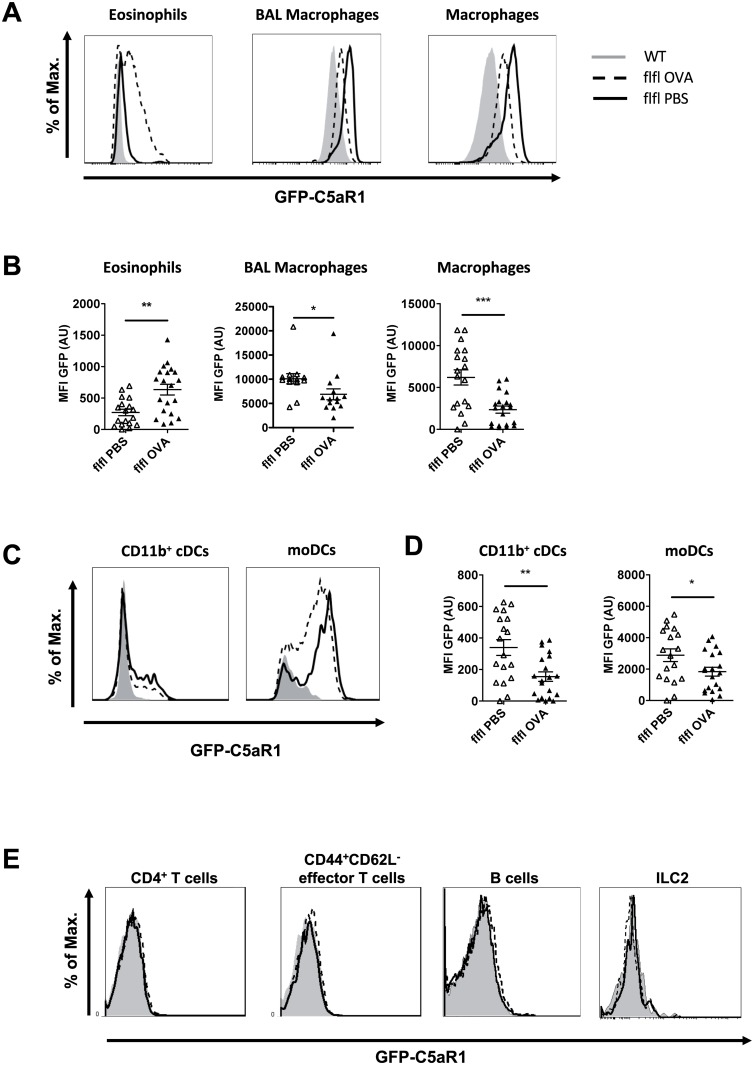
*GFP-C5aR1* expression in innate and adaptive immune cells recruited into the lungs in response to OVA challenge. **(A)** Histograms showing the expression levels of GFP, used as surrogate marker for C5aR1 expression, in lung eosinophils, BAL-derived alveolar and lung macrophages in PBS-treated or OVA-challenged WT and GFP-C5aR1^flox/flox^ animals. (**B**) The corresponding graphs show the relative mean fluorescence intensity (MFI) of the GFP signal in the indicated cell types. Values shown are the mean ± SEM; n = 8–18 per group. **(C)** Histograms showing the expression levels of GFP in CD11b^+^ cDCs and moDCs in PBS-treated or OVA-challenged WT or GFP-C5aR1^flox/flox^ animals. (**D**) The corresponding graphs show the mean fluorescence intensity (MFI) of the GFP signal in the indicated cell types. Values shown are the mean ± SEM; n = 13–16 per group. (**E**) Histograms showing the expression levels of GFP in lung CD4^+^ T cells, CD44^+^CD62L^-^ effector T cells, CD19^+^B220^+^ B cells and ILC2. Histograms are representative of 8–11 animals per group. Grey histogram: GFP signal of WT cells; solid line: GFP signal in cells from PBS-treated GFP-C5aR1^flox/flox^ mice; dashed line: GFP signal in cells from OVA-immunized GFP-C5aR1^flox/flox^ mice.

Next, we compared C5aR1 expression in myeloid cells from BAL, lung and mLNs in response to PBS treatment or OVA immunization. In agreement with our previous findings [15], eosinophils, macrophages and neutrophils homogeneously expressed C5aR1 in the PBS group whereas CD103^+^ cDCs lacked C5aR1 expression (Fig 4A, S2 Fig). The C5aR1 expression was strongest in alveolar macrophages and decreased in lung cells in the order macrophages > neutrophils = moDCs >> CD11b^+^ cDCs > eosinophils (Fig 4A; Table 1). Interestingly, macrophages from the alveolar compartment expressed significantly higher GFP levels than lung macrophages in response to PBS treatment (Table 1). As we found no eosinophils or neutrophils in the BAL of PBS-treated mice (Fig 1C), we could only compare GFP expression in eosinophils or neutrophils from the alveolar and lung tissue compartments following OVA-immunization. While neutrophils from BAL or lung tissue expressed C5aR1 to a similar degree, eosinophils from BAL expressed significantly higher levels of C5aR1 than eosinophils from lung tissue (Table 2). C5aR1 expression in BAL alveolar macrophages and in lung macrophages was significantly down-regulated upon OVA-immunization, (Fig 4A and 4B, Table 1). In contrast to eosinophils, neutrophils and macrophages, we noticed that only a small subpopulation of pulmonary CD11b^+^ cDCs expressed C5aR1 (Fig 4C). While the majority of moDCs expressed C5aR1, some cells stained GFP negative (Fig 4C). During the allergic effector phase, C5aR1 expression was significantly reduced in pulmonary CD11b^+^ cDCs and moDCs as compared with PBS-treated controls (Fig 4D, Table 1).

**Table 1:** Comparison of C5aR1 expression in different myeloid cells in response to PBS.

	MFI BAL	MFI Lung	MFI mLN	p value MFI between compartments
**Eosinophils**	-	270 ± 51^$ $^	-	-
**Neutrophils**	-	3020 ± 319	-	-
**Macrophages**	10097 ± 1066^$^	6201 ± 902^$ $ $^	-	**<0.01**
**CD11b**^**+**^ **cDC**	-	340 ± 50***^$ $^	120 ± 30	**<0.001**
**moDC**	-	2887 ± 401^$^	-	-

Shown are normalized GFP MFI values (mean ± SEM);

^$^ Statistical evaluation of the MFIs of pulmonary eosinophils, neutrophils, macrophages, CD11b^+^ cDCs and moDCs in the PBS vs. OVA treatment groups.

* depicts significant differences between MFIs of lung CD11b^+^ cDCs vs. moDCs in the PBS treatment group;

^$^ p<0.05;

^$ $,^ p<0.01; and

***^, $ $ $,^ p<0.001.

**Table 2:** Comparison of C5aR1 expression in different myeloid cells in response to OVA treatment.

	MFI BAL	MFI Lung	MFI mLN	p value MFI between compartments
**Eosinophils**	1168 ± 152^aa^	634 ± 85	-	**<0.01**
**Neutrophils**	4381 ± 381	3796 ± 485	-	0.35
**Macrophages**	6895 ± 424^bbb^	2355 ± 423	-	**<0.001**
**CD11b**^**+**^ **cDC**	-	155 ± 30^§§§^	132 ± 41	0.64
**moDC**	-	1835 ± 280	666 ± 207	**<0.01**

Shown are normalized GFP MFI values (mean ± SEM);

^a,b^ depict significant differences between the MFIs of ^a^BAL-derived eosinophils vs neutrophils, ^b^eosinophils vs macrophages respectively, in the OVA treated group.

^§^ depicts significant differences between MFIs of lung CD11b^+^ cDCs vs. moDCs in the OVA treatment group.

^aa^ p<0.01; and

^§§§,bbb^ p<0.001.

Importantly, lung tissue eosinophils significantly increased their GFP levels in response to OVA treatment (Fig 4B; left panel; Table 1). In contrast, the inflammatory allergic conditions neither induced C5aR1 expression in CD103^+^ cDCs (S2A Fig) nor did they modulate the expression of C5aR1 in neutrophils (S2B Fig). Interestingly, C5aR1 expression in CD11b^+^ cDCs from mLNs was lower than that in CD11b^+^ cDCs from lung tissue in the PBS-treated group (Table 1). In contrast, the already markedly decreased expression of C5aR1 in pulmonary CD11b^+^ cDCs in response to OVA challenge (as compared to PBS treatment) did not change in CD11^+^ cDCs that were present in the mLNs (Table 2). Of note, the C5aR1 expression of moDCs in mLNs was lower than that of moDCs in the lung (Table 2).

Recent studies reported that CD4^+^ T cells express C5aR1 at steady state and after activation [21, 27]. Previously, we found no GFP signal or C5aR1 mRNA expression in naïve or *in vitro* activated T cells from the spleen [15]. In line with these findings, total and effector CD4^+^ T cells from the lung were negative for the GFP signal in response to PBS treatment or under OVA-driven allergic conditions (Fig 4E). Further pulmonary B cells and ILC2 cells stained C5aR1 negative upon allergic conditions (Fig 4E).

Taken together, our data demonstrate that pulmonary C5aR1 expression is restricted to cells of the myeloid lineage under steady state and allergic conditions. Further, in an established allergic environment, C5aR1 is differentially regulated in macrophages and DCs vs. eosinophils. While C5aR1 is strongly upregulated in eosinophils it is significantly downregulated in macrophages, CD11b^+^ cDCs and moDCs.

### C5aR1 surface expression correlates with GFP expression in eosinophils, macrophages and CD11b^+^ cDCs

**Fig 5:**
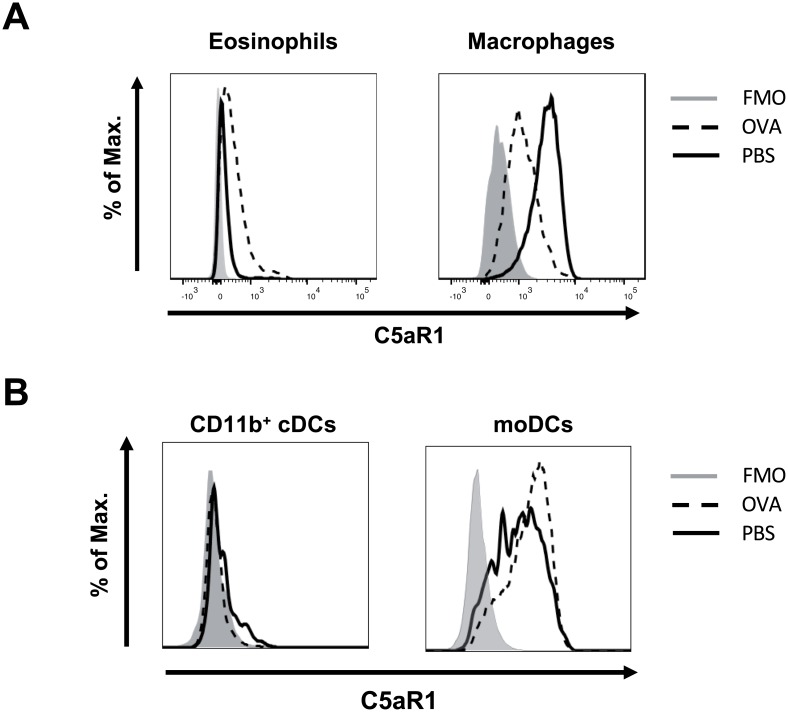
C5aR1 surface expression in innate and adaptive immune cells recruited into the lungs in response to OVA challenge. **(A)** Histograms showing the surface expression levels of C5aR1 in lung eosinophils, and lung macrophages in PBS-treated or OVA-challenged GFP-C5aR1^flox/flox^ animals using C5aR1-specific mAb 20/70. **(B)** Histograms showing the expression levels of GFP in CD11b^+^ cDCs and moDC of PBS-treated or OVA-challenged GFP-C5aR1^flox/flox^ animals. The histograms are representative of three independent experiments.

To assess whether the GFP^+^ cells also express C5aR1 at the cell surface, we counterstained them with a C5aR1-specific antibody. As shown by flow cytometry, naïve lung eosinophils and alveolar macrophages stained homogeneously positive for C5aR1. The specificity was confirmed by comparing the C5aR1 signal in WT and C5aR1^-/-^ mice (S3A Fig). Similarly, the C5aR1 expression followed the GFP expression in lung eosinophils and macrophages (Fig 5A). As observed for the GFP signal (Fig 4A), the surface expression of C5aR1 decreased markedly in response to OVA-immunization but increased significantly on the surface of eosinophils (Fig 5A).

In accordance with our previous findings [15], the pulmonary DC subsets, CD11b^+^ cDCs and moDCs were positive for C5aR1, whereas CD103^+^ cDCs did not express C5aR1 (S3B Fig). Similar to the GFP signal, only a small fraction of CD11b^+^ cDCs expressed C5aR1 on the cell surface (Fig 5B). In response to OVA treatment, C5aR1 surface expression was abrogated in CD11b^+^ cDCs (Fig 5B). The majority of moDCs expressed C5aR1 at the cell surface. In contrast to the GFP expression, we found no clear reduction of the C5aR1 surface signal upon OVA challenge (Fig 5B). Thus, except for moDCs, our data demonstrate that the GFP expression goes along with C5aR1 surface expression.

### Accumulation and C5aR1 expression of DCs and CD4^+^ T cells in mLNs from WT or GFP-C5aR1^flox/flox^ mice

**Fig 6:**
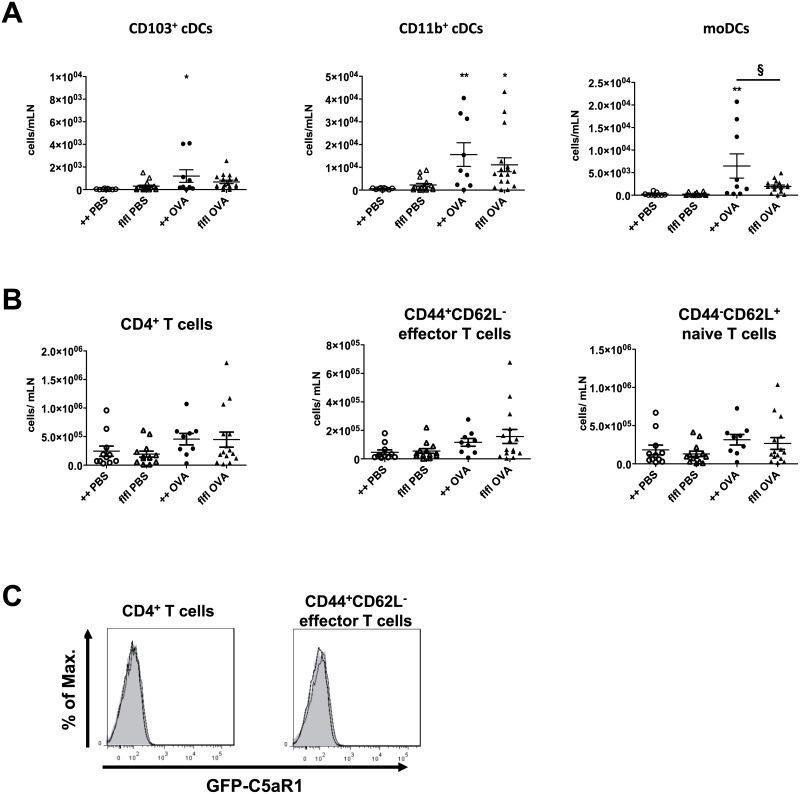
DC and CD4^+^ T cell accumulation and their C5aR1 expression in mLN of WT and GFP-C5aR1^flox/flox^ mice in the allergic effector phase. **(A)** Cell counts of cDC and moDC subsets in mLN of WT or GFP-C5aR1^flox/flox^ mice in response to PBS treatment or OVA immunization; n = 7–15 per group. CD11b^+^ cDC data were analyzed by ANOVA on ranks (n = 7–15). **(B)** Counts of different CD4^+^ T cell subsets in mLN of PBS-treated or OVA-challenged WT or GFP-C5aR1^flox/flox^ animals. Values shown are the mean ± SEM; n = 7–15 per group. * indicates significant differences between PBS and OVA treatment groups; § indicates significant differences between WT and GFP-C5aR1^flox/flox^ OVA-treated groups. * or § p < 0.05; ** p < 0.001. **(C)** GFP signal in CD4^+^ or CD4^+^CD44^+^CD62L^-^ effector T cells from OVA-challenged WT (grey histogram) or GFP-C5aR1^flox/flox^ animals (black line).

In search for mechanisms underlying the decreased accumulation of CD4^+^ T cells in the lung of GFP-C5aR1^flox/flox^ as compared with WT mice, we evaluated the homing of DCs to and the development of effector T cells in the mLNs. We found very low DC numbers in PBS-treated animals. In contrast, we observed a clear increase in CD11b^+^ cDCs and CD103^+^ cDCs in WT and GFP-C5aR1^flox/flox^ mice after OVA challenge, although the number of CD103^+^ cDCs was much lower than the CD11b^+^ cDC numbers (Fig 6A). Also, moDC number increased in response to OVA-immunization. Of note, the number of moDCs in WT was slightly higher than that in GFP-C5aR1^flox/flox^ mice.

T cell numbers in the mLNs were slightly higher after OVA challenge than after PBS treatment. The numbers of total, CD44^+^CD62L^-^ effector or CD44^-^CD62L^+^ naive T cells cells in WT and GFP-C5aR1^flox/flox^ mice mLN were similar (Fig 6B). In line with our observation in T cells from the alveolar and the pulmonary tissue compartments, we found no C5aR1 expression in CD4^+^ T cells from mLNs (Fig 6C). In summary, cDC and CD4^+^ T cell numbers were not altered in GFP-C5aR1^flox/flox^ mice, whereas moDC numbers were slightly decreased. Given that moDCs can prime Th2 effector cell differentiation [13], the decreased number of moDCs in GFP-C5aR1^flox/flox^ mice may explain, at least in part, the decreased pulmonary CD4^+^ T cell number (Fig 3B).

## Discussion

C5a regulates the development of experimental allergic asthma during allergen sensitization and the effector phase through activation of its two cognate receptors C5aR1 and C5aR2 [5]. The C5aR1 expression and activation on defined innate and adaptive immune cells in established allergic asthma is ill-defined. Recently, we demonstrated the expression of C5aR1 in pulmonary eosinophils, neutrophils, macrophages, CD11b^+^ cDCs, and moDCs using a floxed GFP-C5aR1 knock-in mouse [15]. Here, we used this mouse strain as a tool to monitor the expression of C5aR1 in innate and adaptive immune cells in the alveolar space, the lung and the draining lymph nodes during the allergic effector phase.

First, we assessed the allergic phenotype in GFP-C5aR1^flox/flox^ and their WT littermates in an experimental OVA/Alum-driven allergic asthma model. Both strains developed a similar allergic phenotype with comparable airway inflammation and mucus production. However, GFP-C5aR1^flox/flox^ mice were more sensitive to low concentrations of methacholine than their WT controls. These data suggest a change in the contractility of airway smooth muscle cells (ASMs) in the GFP-C5aR1^flox/flox^ animals resulting in airway narrowing at low doses of the bronchoconstrictor. C5aR1 expression in C5aR1^flox/flox^ mice is somewhat lower than in wildtype mice [15]. Further, C5 or C5aR1 targeting is associated with increased airway hyperresponsiveness [3, 7]. Thus, the slightly decreased C5aR1 expression in C5aR1^flox/flox^ mice may account for the increased sensitivity and airway narrowing at low doses of methacholine.

In the lungs of WT and GFP-C5aR1^flox/flox^ mice, we observed a marked increase in macrophages, eosinophils and neutrophils as well as CD11b^+^ and CD103^+^ cDCs and moDCs after the final OVA challenge. The increase in lung cDCs was associated with increased homing of CD11b^+^ and CD103^+^ cDCs to the mLN in WT and C5aR1 reporter mice. Interestingly, the numbers of CD11b^+^ cDCs were about 10-fold higher than that of CD103^+^ cDCs in both strains. In addition, we observed migration of moDCs to the mLN, which was in the range of CD103^+^ cDCs. Of note, the number of moDCs from GFP-C5aR1^flox/flox^ mice that had migrated to the mLNs 24h after the final OVA administration was significantly higher than that of WT mice. A dominant migration of CD11b^+^ cDCs has been described in HDM-driven experimental asthma [13]. However, in contrast to our findings, moDCs were absent 24h after the final allergen challenge and peaked at day 3. The individual role of CD103^+^ and CD11b^+^ cDCs in triggering Th2 immune is still controversial. Important roles for both cDC subtypes have been reported [13, 28, 29]. CD11b^+^ cDCs take up most of the allergen and activate T cells by several costimulatory molecules including CD86 and OX40L or Dectin-2, which are important for Th2/Th17 differentiation and survival [13, 30, 31]. Further, moDCs can prime Th2 immunity [13]. Thus, stronger Th2 priming through the increased number of mLN moDCs in WT mice may account for the higher number of pulmonary CD4^+^ T cells that we found in the lung of WT mice. CD103^+^ cDC and moDCs are the main producers of Th2 homing chemokines CCL17 and CCL22 [13, 32]. The similar numbers of pulmonary CD103^+^ cDCs and moDCs in WT and C5aR1 reporter mice indicate that pulmonary CD4^+^ T cell homing is not affected in C5aR1 reporter mice. In summary, we found that C5aR1 reporter mice exerted a similar allergic phenotype as WT mice with strong AHR, mucus production, airway inflammation and homing of cDCs and moDCs to mLNs. Further, we observed similar inflammation in the lung with comparable numbers of eosinophils, macrophages and neutrophils, CD11b^+^ and CD103^+^ cDCs, moDCs and ILC2, whereas CD4^+^ T cell numbers were lower than in WT controls.

Our previous findings demonstrated that the C5a/C5aR1 signalling axis is instrumental for the development of the allergic asthma phenotype [3, 7]. Previous reports have shown an increase of C5aR1 mRNA in the lung of asthmatic rats [10]. However, the myeloid and/or lymphoid cell types that express the C5aR1 under asthmatic conditions remained unclear. Taking advantage of the C5aR1 reporter mice, we evaluated the C5aR1 expression in myeloid and on lymphoid cells in the airways, the lung and the mLN during the effector phase after the final allergen challenge. Interestingly, C5aR1 expression increased in pulmonary eosinophils. While C5aR1 has been described in eosinophils [33, 34] no data exist about its regulation in lung eosinophils under asthmatic conditions. Eosinophils migrate towards C5a, degranulate [35] and produce reactive oxygen species (ROS) [33]. Thus, the increased C5aR1 expression in lung eosinophils suggests that the C5a/C5aR1 axis plays an important role in the activation of tissue eosinophils. Further, we observed a strong C5aR1 expression in eosinophils accumulating in the airways after final OVA exposure, which was even higher than that of lung tissue eosinophils. Importantly, C5a increases the adhesion of eosinophils through upregulated expression of CD11b [36]. Thus, our data indicate that C5a may play an important role in the extravasation of eosinophils from the circulation into the alveolar space through the regulation of adhesion molecules.

In contrast to eosinophils, C5aR1 expression was high in alveolar macrophages under steady state conditions but decreased significantly after OVA immunization. As macrophage numbers in the airways did not change upon OVA, these data suggest a downregulation of C5aR1 in response to on-going C5a generation in the inflammatory environment of established allergic asthma [3, 4]. Alternatively, the decrease in C5aR1 surface expression could also result from proteolytic C5aR1 cleavage in response to neutrophil-derived serine proteases as described by van den Berg et al. [37], or via an unknown mechanism involving CRP [38]. However, the decrease in surface C5aR1 parallels the decrease in the GFP signal, suggesting down regulation of *C5ar1* mRNA, eventually resulting in decrease of C5aR1 surface expression. Similarly, we observed a decrease in GFP and C5aR1 in tissue-associated macrophages. Recently, C5aR1 has been associated with alveolar macrophage apoptosis in acute lung injury [39]. Further, the C5a/C5aR1 axis contributed to the instability of atherosclerotic lesions by accelerated macrophage apoptosis [40]. Thus, downregulation of C5aR1 on macrophages during the allergic inflammation may limit cell death of alveolar and tissue-associated macrophages to maintain their anti-inflammatory properties [41, 42].

Finally, C5aR1 expression was profoundly regulated in CD11b^+^ cDCs and moDCs. First, C5aR1 was strongly downregulated in CD11b^+^ cDCs that migrated from the lung to the draining LNs under steady state conditions. Previous findings suggest that activation of the C5a/C5aR1 axis on cDCs is important for CD4^+^ T cell proliferation and differentiation [43, 44]. Downregulation of C5aR1 in cDCs trafficking from the lung to draining LNs under steady state conditions may protect from undesired cDC activation. Further, we observed downregulation of the already low C5aR1 expression on pulmonary CD11b^+^ cDCs to almost zero and significant decrease of C5aR1 expression in moDCs after OVA challenge. We and others have previously shown that massive complement occurs in an established allergic environment both in mice [3] and humans [4, 45, 46] resulting in the C3a and C5a generation. Thus, the high concentrations of anaphylatoxins in the lung may account for anaphylatoxin receptor internalization and downregulation on CD11b^+^ cDCs and moDCs. The already decreased C5aR1 expression in moDCs was further downregulated, when the cells migrated to the mLN. Of note, the GFP expression did not match the C5aR1 surface expression determined by a C5aR1-specific antibody. Loss of GFP fluorescence intensity has been observed under low pH conditions (pH<5) [47] which exist in late endosomes or lysosomes [48] and may account for the drop in the MFI. Indeed, intracellular localization of C5aR1 together with C5aR1 has been found in endosomes of human monocytes [49] and CD4^+^ T cells [50] suggesting a redistribution of the C5aR1 protein pool in moDCs under inflammatory allergic conditions.

The expression of C5aR1 in murine T cells at steady state or upon activation is still a matter of debate. Different studies reported C5aR1 expression in resting murine CD4^+^ T cells [21, 27, 51, 52]. In contrast, we and others found no C5aR1 expression in naïve, *in vitro* or *in vivo* activated CD4^+^ T cells, neither at the mRNA nor protein level [15, 20]. Here, we demonstrate that Th2/Th17-differentiated CD4^+^ T cells lack C5aR1 expression during the allergic effector phase. Further, we found no C5aR1 expression in pulmonary B cells and ILC2. Our data confirm that cells from the lymphoid lineage do not express C5aR1, at least not in mice.

In summary, our findings demonstrate a complex regulation of C5aR1 in eosinophils, macrophages CD11b^+^ cDCs and moDCs in the effector phase of allergic asthma. C5aR1 is highly expressed in resident alveolar macrophages as well as neutrophils and eosinophils that migrate into the airways upon allergen challenge. C5aR1 expression on lung tissue macrophages, CD11b^+^ cDCs and moDCs decreases in an established allergic environment but increases in pulmonary eosinophils. The C5aR1 expression in airway or lung tissue neutrophils is high under steady state and allergic conditions. Also, we observed a rather complex pattern of C5aR1 regulation of CD11b^+^ cDCs and moDCs that migrated from the lung into the mLN. In contrast to human CD4^+^ T cells, we neither found C5aR1 expression under steady state nor under allergic conditions, when they differentiated into Th2 or Th17 effector cells. Also, C5aR1 expression was absent in ILC2 and B cells during the allergic effector phase. Our results suggest an important role of the C5a/C5aR1 axis in eosinophil recruitment and/or activation in the effector phase of experimental allergic asthma. Late-onset eosinophilic asthma is a relatively rare form of asthma with frequent severe exacerbations that requires systemic corticoid treatment [53]. C5aR1 may serve as an alternative target or complementary approach to anti-Th2 cytokine antibodies targeting IL-4, IL-5 or IL-13.

## Supporting information

S1 Fig OVA-induced experimental allergic asthma model. WT C5aR1^+/+^ and GFP-C5aR1^flox/flox^ reporter mice were immunized twice i.p. with either PBS/Alum or OVA/Alum at days 0 and 7. Airways were challenged by i.t. administration of 50 μl PBS in the PBS control group or 1.5% OVA solution in PBS in the OVA-immunization group on days 14, 16, 18 and 20. On day 21, lung function was measured, BAL fluid, mediastinal lymph nodes (mLN) and the right lung were harvested for cell isolation, whereas the left lung was used for histology.
(EPS)

S2 Fig Evaluation of C5aR1 expression in neutrophils and CD103^+^ cDCs. **(A)** Histograms showing the expression levels of GFP, used as surrogate marker for C5aR1 expression, in lung neutrophils in PBS-treated or OVA-challenged GFP-C5aR1^flox/flox^ animals. The corresponding graph on the right shows the mean fluorescence intensity (MFI) of the GFP signal in neutrophils. Values shown are the mean ± SEM; n = 8–18 per group. (**B**) Histograms showing the expression levels of GFP, used as surrogate marker for C5aR1 expression, in lung CD103+ cDCs in PBS-treated or OVA-challenged GFP-C5aR1^flox/flox^ animals.
(EPS)

S3 Fig Surface expression of C5aR1. Cells isolated from WT and C5aR1^-/-^ mice under steady state conditions were stained for C5aR1 (CD88) as described in material and methods. **(A/B)** C5aR1 surface staining on eosinophils and tissue-associated alveolar macrophages **(A)** as well as different DC subsets **(B)** as described in Fig 2D. Data are representative of two independent cell isolations. Histograms show fluorescence intensity in cells from WT (solid line) and C5aR1^-/-^ mice (dashed line).
(EPS)

## References

[1] Alfven T, Braun-Fahrlander C, Brunekreef B, von Mutius E, Riedler J, Scheynius A, et al Allergic diseases and atopic sensitization in children related to farming and anthroposophic lifestyle—the PARSIFAL study. Allergy. 2006;61:414–421. 10.1111/j.1398-9995.2005.00939.x 16512802

[2] Maruo K, Akaike T, Ono T, Okamoto T, Maeda H. Generation of anaphylatoxins through proteolytic processing of C3 and C5 by house dust mite protease. J Allergy Clin Immunol. 1997;100:253–260. 9275149 10.1016/s0091-6749(97)70233-1

[3] Kohl J, Baelder R, Lewkowich IP, Pandey MK, Hawlisch H, Wang L, et al A regulatory role for the C5a anaphylatoxin in type 2 immunity in asthma. J Clin Invest. 2006;116:783–796. 10.1172/JCI26582 16511606 PMC1386108

[4] Krug N, Tschernig T, Erpenbeck VJ, Hohlfeld JM, Kohl J. Complement factors C3a and C5a are increased in bronchoalveolar lavage fluid after segmental allergen provocation in subjects with asthma. Am J Respir Crit Care Med. 2001;164:1841–1843. 10.1164/ajrccm.164.10.2010096 11734433

[5] Schmudde I, Laumonnier Y, Kohl J. Anaphylatoxins coordinate innate and adaptive immune responses in allergic asthma. Semin Immunol. 2013;25:2–11. 10.1016/j.smim.2013.04.009 23694705

[6] Drouin SM, Sinha M, Sfyroera G, Lambris JD, Wetsel RA. A protective role for the fifth complement component (c5) in allergic airway disease. Am J Respir Crit Care Med. 2006;173:852–857. 10.1164/rccm.200503-334OC 16439722 PMC2662907

[7] Karp CL, Grupe A, Schadt E, Ewart SL, Keane-Moore M, Cuomo PJ, et al Identification of complement factor 5 as a susceptibility locus for experimental allergic asthma. Nat Immunol. 2000;1:221–226. 10.1038/79759 10973279

[8] Lajoie S, Lewkowich IP, Suzuki Y, Clark JR, Sproles AA, Dienger K, et al Complement-mediated regulation of the IL-17A axis is a central genetic determinant of the severity of experimental allergic asthma. Nat Immunol. 2010;11:928–935. 10.1038/ni.1926 20802484 PMC2943538

[9] Zhang X, Lewkowich IP, Kohl G, Clark JR, Wills-Karp M, Kohl J. A protective role for C5a in the development of allergic asthma associated with altered levels of B7-H1 and B7-DC on plasmacytoid dendritic cells. J Immunol. 2009;182:5123–5130. 10.4049/jimmunol.0804276 19342693 PMC2923383

[10] Abe M, Shibata K, Akatsu H, Shimizu N, Sakata N, Katsuragi T, et al Contribution of anaphylatoxin C5a to late airway responses after repeated exposure of antigen to allergic rats. J Immunol. 2001;167:4651–4660. 11591795 10.4049/jimmunol.167.8.4651

[11] Baelder R, Fuchs B, Bautsch W, Zwirner J, Kohl J, Hoymann HG, et al Pharmacological targeting of anaphylatoxin receptors during the effector phase of allergic asthma suppresses airway hyperresponsiveness and airway inflammation. J Immunol. 2005;174:783–789. 15634899 10.4049/jimmunol.174.2.783

[12] Hawlisch H, Kohl J. Complement and Toll-like receptors: key regulators of adaptive immune responses. Mol Immunol. 2006;43:13–21. 10.1016/j.molimm.2005.06.028 16019071

[13] Plantinga M, Guilliams M, Vanheerswynghels M, Deswarte K, Branco-Madeira F, Toussaint W, et al Conventional and monocyte-derived CD11b(+) dendritic cells initiate and maintain T helper 2 cell-mediated immunity to house dust mite allergen. Immunity. 2013;38:322–335. 10.1016/j.immuni.2012.10.016 23352232

[14] Hoffmann F, Ender F, Schmudde I, Lewkowich IP, Kohl J, Konig P, et al Origin, Localization, and Immunoregulatory Properties of Pulmonary Phagocytes in Allergic Asthma. Front Immunol. 2016;7:107 10.3389/fimmu.2016.00107 27047494 PMC4803735

[15] Karsten CM, Laumonnier Y, Eurich B, Ender F, Broker K, Roy S, et al Monitoring and cell-specific deletion of C5aR1 using a novel floxed GFP-C5aR1 reporter knock-in mouse. J Immunol. 2015;194:1841–1855. 10.4049/jimmunol.1401401 25589074

[16] Nakano H, Moran TP, Nakano K, Gerrish KE, Bortner CD, Cook DN. Complement receptor C5aR1/CD88 and dipeptidyl peptidase-4/CD26 define distinct hematopoietic lineages of dendritic cells. J Immunol. 2015;194:3808–3819. 10.4049/jimmunol.1402195 25769922 PMC4390500

[17] Solomkin JS, Jenkins MK, Nelson RD, Chenoweth D, Simmons RL. Neutrophil dysfunction in sepsis. II. Evidence for the role of complement activation products in cellular deactivation. Surgery. 1981;90:319–327. 7256544

[18] Gerard NP, Hodges MK, Drazen JM, Weller PF, Gerard C. Characterization of a receptor for C5a anaphylatoxin on human eosinophils. J Biol Chem. 1989;264:1760–1766. 2912983

[19] Skokowa J, Ali SR, Felda O, Kumar V, Konrad S, Shushakova N, et al Macrophages induce the inflammatory response in the pulmonary Arthus reaction through G alpha i2 activation that controls C5aR and Fc receptor cooperation. J Immunol. 2005;174:3041–3050. 15728518 10.4049/jimmunol.174.5.3041

[20] Dunkelberger J, Zhou L, Miwa T, Song WC. C5aR expression in a novel GFP reporter gene knockin mouse: implications for the mechanism of action of C5aR signaling in T cell immunity. J Immunol. 2012;188:4032–4042. 10.4049/jimmunol.1103141 22430734 PMC3324670

[21] Strainic MG, Liu J, Huang D, An F, Lalli PN, Muqim N, et al Locally produced complement fragments C5a and C3a provide both costimulatory and survival signals to naive CD4+ T cells. Immunity. 2008;28:425–435. 10.1016/j.immuni.2008.02.001 18328742 PMC2646383

[22] Tschernig T, Kiafard Z, Dibbert C, Neumann D, Zwirner J. Use of monoclonal antibodies to assess expression of anaphylatoxin receptors in rat and murine models of lung inflammation. Exp Toxicol Pathol. 2007;58:419–425. 10.1016/j.etp.2007.03.004 17544263

[23] Zhang X, Schmudde I, Laumonnier Y, Pandey MK, Clark JR, Konig P, et al A critical role for C5L2 in the pathogenesis of experimental allergic asthma. J Immunol. 2010;185:6741–6752. 10.4049/jimmunol.1000892 20974988

[24] Schmudde I, Strover HA, Vollbrandt T, Konig P, Karsten CM, Laumonnier Y, et al C5a receptor signalling in dendritic cells controls the development of maladaptive Th2 and Th17 immunity in experimental allergic asthma. Mucosal Immunol. 2013;6:807–825. 10.1038/mi.2012.119 23212198

[25] Monticelli LA, Sonnenberg GF, Abt MC, Alenghat T, Ziegler CG, Doering TA, et al Innate lymphoid cells promote lung-tissue homeostasis after infection with influenza virus. Nat Immunol. 2011;12:1045–1054. 10.1031/ni.2131 21946417 PMC3320042

[26] Halim TY, Steer CA, Matha L, Gold MJ, Martinez-Gonzalez I, McNagny KM, et al Group 2 innate lymphoid cells are critical for the initiation of adaptive T helper 2 cell-mediated allergic lung inflammation. Immunity. 2014;40:425–435. 10.1016/j.immuni.2014.01.011 24613091 PMC4210641

[27] Strainic MG, Shevach EM, An F, Lin F, Medof ME. Absence of signaling into CD4(+) cells via C3aR and C5aR enables autoinductive TGF-beta1 signaling and induction of Foxp3(+) regulatory T cells. Nat Immunol. 2013;14:162–171. 10.1038/ni.2499 23263555 PMC4144047

[28] Zhou Q, Ho AW, Schlitzer A, Tang Y, Wong KH, Wong FH, et al GM-CSF-licensed CD11b+ lung dendritic cells orchestrate Th2 immunity to Blomia tropicalis. J Immunol. 2014;193:496–509. 10.4049/jimmunol.1303138 24943219

[29] Nakano H, Free ME, Whitehead GS, Maruoka S, Wilson RH, Nakano K, et al Pulmonary CD103(+) dendritic cells prime Th2 responses to inhaled allergens. Mucosal Immunol. 2012;5:53–65. 10.1038/mi.2011.47 22012243 PMC3697034

[30] Jember AG, Zuberi R, Liu FT, Croft M. Development of allergic inflammation in a murine model of asthma is dependent on the costimulatory receptor OX40. J Exp Med. 2001;193:387–392. 11157058 10.1084/jem.193.3.387 PMC2195923

[31] Norimoto A, Hirose K, Iwata A, Tamachi T, Yokota M, Takahashi K, et al Dectin-2 promotes house dust mite-induced T helper type 2 and type 17 cell differentiation and allergic airway inflammation in mice. Am J Respir Cell Mol Biol. 2014;51:201–209. 10.1165/rcmb.2013-0522OC 24588637

[32] Ortiz-Stern A, Kanda A, Mionnet C, Cazareth J, Lazzari A, Fleury S, et al Langerin+ dendritic cells are responsible for LPS-induced reactivation of allergen-specific Th2 responses in postasthmatic mice. Mucosal Immunol. 2011;4:343–353. 10.1038/mi.2010.73 21048704

[33] Elsner J, Oppermann M, Kapp A. Detection of C5a receptors on human eosinophils and inhibition of eosinophil effector functions by anti-C5a receptor (CD88) antibodies. Eur J Immunol. 1996;26:1560–1564. 10.1002/eji.1830260723 8766561

[34] Haviland DL, McCoy RL, Whitehead WT, Akama H, Molmenti EP, Brown A, et al Cellular expression of the C5a anaphylatoxin receptor (C5aR): demonstration of C5aR on nonmyeloid cells of the liver and lung. J Immunol. 1995;154:1861–1869. 7836770

[35] Goetzl EJ. Modulation of human eosinophil polymorphonuclear leukocyte migration and function. Am J Pathol. 1976;85:419–436. 793410 PMC2032558

[36] Fujiu T, Kato M, Kimura H, Tachibana A, Suzuki M, Nako Y, et al Cellular adhesion is required for effector functions of human eosinophils via G-protein coupled receptors. Ann Allergy Asthma Immunol. 2002;89:90–98. 10.1016/S1081-1206(10)61917-5 12141728

[37] van den Berg CW, Tambourgi DV, Clark HW, Hoong SJ, Spiller OB, McGreal EP. Mechanism of neutrophil dysfunction: neutrophil serine proteases cleave and inactivate the C5a receptor. J Immunol. 2014;192:1787–1795. 10.4049/jimmunol.1301920 24446515

[38] Unnewehr H, Rittirsch D, Sarma JV, Zetoune F, Flierl MA, Perl M, et al Changes and regulation of the C5a receptor on neutrophils during septic shock in humans. J Immunol. 2013;190:4215–4225. 10.4049/jimmunol.1200534 23479227

[39] Hu R, Chen ZF, Yan J, Li QF, Huang Y, Xu H, et al Complement C5a exacerbates acute lung injury induced through autophagy-mediated alveolar macrophage apoptosis. Cell Death Dis. 2014;5:e1330 10.1038/cddis.2014.274 25032853 PMC4123068

[40] Wezel A, de Vries MR, Lagraauw HM, Foks AC, Kuiper J, Quax PH, et al Complement factor C5a induces atherosclerotic plaque disruptions. J Cell Mol Med. 2014;18:2020–2030. 10.1111/jcmm.12357 25124749 PMC4244017

[41] Westphalen K, Gusarova GA, Islam MN, Subramanian M, Cohen TS, Prince AS, et al Sessile alveolar macrophages communicate with alveolar epithelium to modulate immunity. Nature. 2014;506:503–506. 10.1038/nature12902 24463523 PMC4117212

[42] Soroosh P, Doherty TA, Duan W, Mehta AK, Choi H, Adams YF, et al Lung-resident tissue macrophages generate Foxp3+ regulatory T cells and promote airway tolerance. J Exp Med. 2013;210:775–788. 10.1084/jem.20121849 23547101 PMC3620360

[43] Peng Q, Li K, Patel H, Sacks SH, Zhou W. Dendritic cell synthesis of C3 is required for full T cell activation and development of a Th1 phenotype. J Immunol. 2006;176:3330–3341. 16517700 10.4049/jimmunol.176.6.3330

[44] Peng Q, Li K, Wang N, Li Q, Asgari E, Lu B, et al Dendritic cell function in allostimulation is modulated by C5aR signaling. J Immunol. 2009;183:6058–6068. 10.4049/jimmunol.0804186 19864610

[45] Humbles AA, Lu B, Nilsson CA, Lilly C, Israel E, Fujiwara Y, et al A role for the C3a anaphylatoxin receptor in the effector phase of asthma. Nature. 2000;406:998–1001. 10.1038/35023175 10984054

[46] Nakano Y, Morita S, Kawamoto A, Suda T, Chida K, Nakamura H. Elevated complement C3a in plasma from patients with severe acute asthma. J Allergy Clin Immunol. 2003;112:525–530. 13679811 10.1016/s0091-6749(03)01862-1

[47] Ishii M, Kunimura JS, Jeng HT, Penna TC, Cholewa O. Evaluation of the pH- and thermal stability of the recombinant green fluorescent protein (GFP) in the presence of sodium chloride. Appl Biochem Biotechnol. 2007;137–140:555–571. 10.1007/s12010-007-9079-6 18478416

[48] Sorkin A, Von Zastrow M. Signal transduction and endocytosis: close encounters of many kinds. Nat Rev Mol Cell Biol. 2002;3:600–614. 10.1038/nrm883 12154371

[49] Croker DE, Halai R, Fairlie DP, Cooper MA. C5a, but not C5a-des Arg, induces upregulation of heteromer formation between complement C5a receptors C5aR and C5L2. Immunol Cell Biol. 2013;91:625–633. 10.1038/icb.2013.48 24060963

[50] Arbore G, West EE, Spolski R, Robertson AA, Klos A, Rheinheimer C, et al T helper 1 immunity requires complement-driven NLRP3 inflammasome activity in CD4(+) T cells. Science. 2016;352:aad1210 10.1126/science.aad1210 27313051 PMC5015487

[51] Kwan WH, van der Touw W, Paz-Artal E, Li MO, Heeger PS. Signaling through C5a receptor and C3a receptor diminishes function of murine natural regulatory T cells. J Exp Med. 2013;210:257–268. 10.1084/jem.20121525 23382542 PMC3570105

[52] van der Touw W, Cravedi P, Kwan WH, Paz-Artal E, Merad M, Heeger PS. Cutting edge: Receptors for C3a and C5a modulate stability of alloantigen-reactive induced regulatory T cells. J Immunol. 2013;190:5921–5925. 10.4049/jimmunol.1300847 23690475 PMC3679341

[53] de Groot JC, Ten Brinke A, Bel EH. Management of the patient with eosinophilic asthma: a new era begins. ERJ Open Res. 2015;1. 10.1183/23120541.00024-2015 PMC5005141 27730141

